# Research on quantum cognition in autonomous driving

**DOI:** 10.1038/s41598-021-04239-y

**Published:** 2022-01-07

**Authors:** Qingyuan Song, Wen Wang, Weiping Fu, Yuan Sun, Denggui Wang, Zhiqiang Gao

**Affiliations:** 1 Xi’an University of Technology, Xi’an, China; 2 Xi’an International University, Xi’an, China

**Keywords:** Electrical and electronic engineering, Mechanical engineering

## Abstract

Autonomous vehicles for the intention of human behavior of the estimated traffic participants and their interaction is the main problem in automatic driving system. Classical cognitive theory assumes that the behavior of human traffic participants is completely reasonable when studying estimation of intention and interaction. However, according to the quantum cognition and decision theory as well as practical traffic cases, human behavior including traffic behavior is often unreasonable, which violates classical cognition and decision theory. Based on the quantum cognitive theory, this paper studies the cognitive problem of pedestrian crossing. Through the case analysis, it is proved that the Quantum-like Bayesian (QLB) model can consider the reasonability of pedestrians when crossing the street compared with the classical probability model, being more consistent with the actual situation. The experiment of trajectory prediction proves that the QLB model can cover the edge events in interactive scenes compared with the data-driven Social-LSTM model, being more consistent with the real trajectory. This paper provides a new reference for the research on the cognitive problem of intention on bounded rational behavior of human traffic participants in autonomous driving.

## Introduction

On October 11, 2020, Baidu, the search engine of China announced that its autonomous taxi service will be fully into markets in Beijing. Citizens can directly order autonomous taxi for free without reservation at dozens of autonomous taxi stations in Beijing Economic and Technological Development Zone, Haidian District and Shunyi District. After experiencing for the first time, some passengers reported that other traffic participants must obey the traffic rules when the autonomous vehicle is on the road, otherwise the autonomous vehicle cannot move at all, because some pedestrians and non-motor vehicles do not obey the traffic rules, especially their crossing the traffic lights. Autonomous vehicles can only stop after watching there are some pedestrians and non-motor vehicles around^1^.

For a long time in the future, autonomous vehicles will inevitably share urban roads with human traffic participants^2^. In order to drive safely and efficiently in this complex traffic surrounding, autonomous driving vehicles need to correctly estimate the behavioral intention of human traffic participants and interact with human traffic participants naturally just like human driving vehicles^3,4^. The behavior of human traffic participants and their interactions are very random in the real world actually. Osamu proposed that such randomness is characterized by obvious uncertainty and irrationality^5^. The “long tail” problem of autonomous driving includes various fragmented scenarios, extreme situations and unpredictable human behavior. This is related to the unreasonable behavior intention and uncertainty^6^, which needs to be studied by correct and effective cognitive and decision theory.

Behavioral intention reveals the purpose of taking a certain action or achieving a certain goal, which is understood as the cause of human actions. Human intentions are internal processes and can generally be inferred by observing the actions they produce^7,8^. At present, the main research methods on intention estimation and behavior prediction include: traditional machine learning based on classical probability (model driven) and deep learning (data driven)^9,10^.

Traditional machine learning methods generally regard the evolution process of traffic participants’ behavior as having the property of Markov decision process (MDP)^11^, and usually use hidden Markov model (HMM), dynamic Bayesian network (DBN) and other methods to infer intent. Due to the high nonlinearity of vehicle and pedestrian behavior intention, movement trajectory and their interaction, as well as the diversity of human traffic participants, it is difficult for traditional model-driven machine learning methods to achieve satisfactory accuracy in intention estimation and behavior as well as trajectory prediction in the far future. At the same time the deep learning method based on data driven, such as long short-Term Memory network (LSTM), needs a large amount of data to support. If the data quantity is not enough, it will cause over fitting, and this method cannot explain the causal relationship between^12^, the myriad of change of data and automated driving scene. Real world scenario is difficult to complete by using the method of artificial statistics received. And the automatic driving scenario requires strong logic and interpretability^13^.

What is gratifying is that quantum theory originated in the field of microscopic physics has been extended in the past two decades and it made great progress in many non-physical and macro fields such as cognition, decision making, information, communication, computing, etc. It has not only formed an increasingly mature theoretical system, but also been increasingly widely applied^14^. In particular, the initial quantum inkling in the field of mobile robots, being most closely related to unmanned driving technology^14^, allows us to see the potential and possibility of applying quantum theory to solve the cognitive problems of autonomous driving. Quantum theory provides a new way to study the uncertain behavior (including irrational behavior) of human traffic participants and their interaction. How to correctly understand the uncertain behavior and interaction of human traffic participants based on quantum theory, and how to make correct interactive behavior decisions based on this is the focus of this paper waiting to explore and solve.

In this paper, the case of pedestrian crossing is analyzed through the Quantum-like Bayes (QLB) method. Feasibility of this method is proved through experiments. At the same time, it is proved that the use of quantum cognitive theory to predict the intention of human behavior can be interpreted in the field of autonomous driving, which is not achieved by other methods at present. This paper is the first attempt to apply quantum cognition theory into autonomous driving.

## Related work

### Model-driven machine learning to predict human behavioral intentions

Many researches focus on the estimation of intention behind human driving and behavior prediction. Liu^9^ proposed a driving intention prediction method for autonomous vehicles based on HMM, HMM trained with continuous mobility features can provide higher prediction accuracy when predicting driving intention. Zhang^15^ proposed a lane change intention estimation framework based on Gauss-Hidden Markov Mixture Model (GMM + HMM). Liu^16^ proposed a semi-Markov model based on nonlinear polynomial regression and recursive hidden model (R-HSMM), which can identify driver intentions earlier than common methods and better adapt to long-term continuous state. Considering the influence of interaction between vehicles on behavior prediction, Zhang^10^ proposed an interactive prediction and recognition based on game theory and GMM + HMM model to predict the intention of other vehicles and identify their behaviors. Wang^17^ proposed an intention reasoning algorithm based on interactive games to solve the interactive “double-blind” intention reasoning problem between two agents. Lefkopoulos^18^ proposed a multi-vehicle motion prediction scheme based on interactive multi-model and Kalman filter (IMM-KF), which was able to predict the collision free and interactive sensing trajectories of multiple traffic participants. Compared with other vehicle intention estimation, intention estimation and behavior (trajectory) prediction of pedestrians and cyclists are still challenging^19–21^. Sun^22^ proposed a multi-agent hybrid dynamic Bayesian network (MDBN) integration framework, which simultaneously estimated and predicted the interaction intention and trajectory between heterogeneous traffic participants (vehicle and pedestrian). Li^23^ combined DBN reasoning and sequence learning through an adaptive weighting strategy to predict pedestrian crossing behavior tracks at random intersections. Rudenko^24^ described the long-term target oriented multi-agent global motion prediction task as an MDP programming problem, and used social force model and random walk strategy to predict the interactive motions of multiple agents. Current researches on the behavioral intentions of human traffic participants based on machine learning are implicitly based on the assumption of complete rationality and mutual independence, and all of them adopt the cognitive theory based on classical probability, obviously being inconsistent with the traffic scenes in reality.

### Prediction of human behavior intentions based on data-driven deep learning methods

In recent years, the implementation of human traffic participant intention estimation and trajectory prediction based on data-driven deep learning methods has attracted more and more scholars' attention. Xu et al.^25^ summarized the literature on the problem of dynamic decision making with the data obtained by roadside units, and proposed that the deep learning algorithm would cause over-fitting phenomenon, and DRL should be combined with other algorithms to solve this open problem; Azidine Guezzaz et al.^26^ proposed a data-driven classifier to monitor traffic. However, due to the large dimension and heterogeneity of data, this method has certain limitations. Nait Malek et al.^27^ used LSTM method to predict the speed of traffic participants and proved that the method is obtained with certain accuracy.

Long Short-Term Memory Network (LSTM) is a special cyclic neural network (RNN), which shows strong ability of information mining and deep representation when dealing with intention estimation and trajectory prediction with temporal characteristics, and has been widely used in behavior prediction of vehicles^12,28,29^ and pedestrians^21,30^. However, due to the deficiencies of RNN in modeling spatial relations (such as vehicle spatial interaction and driving scene context), additional methods are often used to compensate for the weaknesses of RNN or the deep neural network framework different from RNN framework^12^. Social spatiotemporal graph convolutional neural network^31–35^ and unsupervised learning method^13^ are widely used in recent years. Due to the low sample efficiency of purely data-driven methods, the interaction between traffic participants and their environment cannot be effectively described through statistical observations alone. As a result, the generalization ability or interpretability in the prediction is in a low way.

### The feasibility of quantum cognitive theory in predicting human behavior intentions

Most studies only regard traffic participants as moving objects without cognitive ability and assume that their behavior is reasonable^30,36^, and most of them are based on classical probabilistic machine learning theories (HMM, DBN, MDP, GP, etc.) or data-driven deep learning methods (RMN, etc.). Inference, estimation and prediction only from the perspective of ontology or phenomenology still lack a powerful and convincing cognitive model to fully and accurately describe human behavior^37^. In fact, human behavior is often peculiar rather than completely reasonable and it generally does not satisfy the Markov properties or known parameter distributions based on classical probability theory^38^. There has been evidence showing that human bounded rational behaviors, especially irrational behaviors, including human decision-making behaviors in traffic environment, tend to deviate from the expectation of behavior theory based on classical probability^11,22,39^, and thus may become potential risks of autonomous and safe driving. Non-classical probabilistic cognitive theories (e.g., quantum cognitive theory^40^) are required for correct reasoning, estimation, and prediction.

Quantum mechanics is the greatest discovery of the last century, greatly facilitating modern science and technology and later become the theoretical guidance of new science and technology. Researchers in the field of cognition have found that interference and entanglement in the quantum mechanics and the interaction of human cognition have many similar characteristics, and they build the mathematical expression of quantum mechanics. Quantum probability is introduced into the cognitive domain, trying to take advantage of unique features to build cognitive model of quantum mechanics for the purpose of explaining the difficult problems in the field of human cognition that classical probability cannot explain. Quantum cognitive decision theory based on quantum probability was gradually born^41^. Quantum logic was proposed by the famous mathematician Von Neumann, who defined events as a subspace in the Hilbert space^41^. In this way, quantum probability does not need to be constrained by Boolean logic laws, such as the law of total probability. Therefore, quantum decision theory can allow the existence of events violating the law of full probability. Busemeyer and Bruza pointed out that quantum logic is actually a generalized Boolean logic, which is not limited by many constraints in Boolean logic, and it has greater flexibility and randomness, being more conducive to explaining human judgments and decisions^42^. In the past 10 years, quantum cognitive decision theory has made a series of breakthroughs in human cognition and it has been recognized as a new way to explore human cognitive science^43^. The quantum cognitive decision theory (such as Quantum-like Bayesian (QLB) theory^42^, quantum game theory^42^, etc.) produced by the combination of quantum probability and classical machine learning theory (MDP, POMDP, DBN, HMM, etc.) provides a more advanced and effective theoretical tool for the study of cognitive decision making of autonomous driving systems.

In conclusion, there is a lack of systematic approaches to autonomous driving cognition that take the irrational behaviors of human traffic participants and their interactions into account. Although quantum cognitive theory has made great progress in recent years, providing a new method for the study of autonomous driving cognitive problems considers the interaction of human traffic participants' behaviors (including irrational behaviors).There is no case study applied to the field of autonomous driving. In this paper, the case about pedestrian crossing is analyzed by the Quantum-like Bayesian (QLB) method, which is the first attempt to apply the quantum cognitive theory into automatic driving.

## Method

### Classical probability and quantum probability

#### Operational differences between classical probability and quantum probability

Let's assume that a system has attribute A, and its value can be up and down, In addition, the system also has attribute B, and its value can be left and right. The biggest difference between quantum probability and classical probability is that there are incompatible attribute pairs, that is, two attributes cannot be measured at the same time. Correspondingly, if two attributes can be measured at the same time, they constitute a compatible attribute pair. For the measurement of an attribute, quantum probability and classical probability will get exactly the same result. Furthermore, for compatible attribute pairs, there is still no difference between quantum probability and classical probability. In other words, the compatible attribute operation in quantum probability has been able to cover all the contents of classical probability theory. However, for incompatible attribute pairs, many classical probability algorithms are no longer valid. The properties of classical probability system can be found in the measurement of compatible attributes of quantum probability, but conversely, the incompatible attributes in quantum probability have special properties, so it can be said that quantum probability contains more probability operation systems than classical probability.

#### Collapse, entanglement and separation effects in quantum probability

Bit is the basic unit of classical computation and information storage in classical information field,Bit is a kind of binary logic, which can only be in one state, namely 0 or 1. The corresponding qubit^44^ is the basic unit of information storage in the field of quantum computing and quantum information,the two possible states of qubit are $\left|0\right.\rangle$ and $\left|1\right.\rangle$, the classical bit can only be in one of 0 or 1, that is, either 0 or 1, while the state of qubit can be any intermediate state of two ground States, that is, linear combination, called superposition state, which means:

1 $$\left|\psi \right.\rangle =\alpha \left|0\right.\rangle +\beta \left|1\right.\rangle$$

where α and β are complex numbers and satisfy the normalization condition ${| \alpha |}^{2}+{| \beta |}^{2} = 1$. In quantum mechanics, if $\left|\psi \right.\rangle$ is measured,the superimposed state will collapse, with a probability of ${| \alpha |}^{2}$ to the state $\left|0\right.\rangle$ and ${| \beta |}^{2}$ to the state $\left|1\right.\rangle$, the final measurement result can only get one of $\left|0\right.\rangle$ or $\left|1\right.\rangle$, so it is impossible to judge the accurate state of qubits by measurement, that is, the exact values of α and β cannot be obtained.

Different from classical probability, quantum entanglement still exists in quantum probability. Quantum entanglement is a phenomenon that quantum influences each other in a system composed of two or more quanta, which is extended to nonlocal association among subsystems in a composite system, and occupies an extremely important position in quantum information theory^45^. In a composite system, if there are interactions among subsystems, there will be entanglement among subsystems. If there is entangled state in a composite system, the operation of one subsystem will definitely affect the other subsystems. Quantum entanglement is of great significance in quantum game theory, Eisert^46^ and others have proved that when the entangled state in quantum game is zero, the game is a classical game, when the entangled state is maximum, the game is completely quantized, and its strategy combination will be superior to the classical game.

Quantum probability can also explain the separation effect that classical probability cannot explain. The principle of determining events is a basic principle of classical probability, that is, decision makers will choose to execute action A when they know that event E occurs, but they still choose to execute action A when they do not know that event E occurs. However, in the real world, decision makers will be in a hesitant state whether to execute action A when they do not know that event E occurs, which violates the classical probability. Tversky and Shafir call this strange decision-making phenomenon separation effect^47^. Pothos and Busemeyer proved that quantum probability can explain the separation effect^48^.

To sum up, quantum probability has wider physical meaning and properties than classical probability. Measurement is an important way to transform the illusory world of quantum into the real world, and human consciousness itself is transforming various possibilities into reality. This makes many scientists and philosophers think that quantum probability can not only describe the microscopic particle world, but also describe human consciousness and cognitive behavior^14^.

### Classical Bayesian Model (CBM) and Quantum-like Bayesian (QLB) Model

#### Classical Bayesian Model (CBM))

The CBM is a directed acyclic graph structure, in which each node represents a different random variable in a specific domain, and each edge represents the direct impact of the source node on the target node. The core of the Bayesian network is that nodes are directly dependent on their parents. Once the value of the parent node is known, any information directly or indirectly related to the parent node or other ancestor nodes cannot affect the belief in its value. Nevertheless, information about its offspring can change the way people think about it^49^. This leads to conditional independence, which leads to the Markov hypothesis.

Markov hypothesis can be defined as follows: Make $X=\{{X}_{1},{X}_{2},...{X}_{N}\}$ is a set of $N$ random variables of a Bayesian Network structure,making Parent(${X}_{i}$) is the Parent of the random variable ${X}_{i}$ and making Non-Descendant (${X}_{i}$) is a variable in the graph that is not descendant of ${X}_{i}$, then, the Markov hypothesis states that given its Parent node, each variable ${X}_{i}$ is independent of its non-descendant, namely:

2 $${X}_{i}\perp NonDescendant({X}_{i})|Parents\left({X}_{i}\right)$$

Each node Xi is conditionally independent from the non-descendant node of a given parent node, so we can use Eq. (3) to factor the Bayesian Network, where the parameter σ corresponds to the normalization factor:

3 $$Pr(X_{1} , \ldots ,X_{n} ) = \sigma \prod\limits_{{i = 1}}^{n} {Pr\left( {X_{i} |Parents\left( {X_{i} } \right)} \right)}$$

The Bayesian Network represents an arbitrary complete joint distribution in a concise way, which has certain advantages. Equation (3) is used instead of calculating the complete joint distribution, and the joint distribution of random variables can be calculated from the probability distribution of the parent node.

Related to the Bayesian Network is the concept of conditional independence. Two random variables X and Y are conditionally independent, and let's say that the third random variable Z is conditional independent if and only if they are independent in a conditional probability distribution, let's say that Z, X and Y are conditionally independent. If and only if, given any value of Z, the probability distribution of X is the same for all values of Y, and similarly, the probability distribution of Y is the same for all values of X. This means that an independent statement of a random variable is a general quantification of all possible values of the random variable^49^. Therefore, a probability distribution satisfies (X⊥Y|Z) if and only if:

4 $${\text{Pr}}\;(X,\left. Y \right|Z) = {\text{Pr}}\;(\left. X \right|Z)\;{\text{Pr}}\;(\left. Y \right|Z)$$

#### Quantum-like Bayesian (QLB) Model

QLB Network can also be defined with a directed acyclic graph structure, where each node represents a different quantum random variable, and each edge represents the direct impact of the source node on the target node. The definition of QLB network is the same as that of classical network, except that the actual probability value is replaced by the complex probability amplitude^49^.

As a classical probability theory, a random variable is a function that maps a set of values contained in a sample space to real numbers. In quantum mechanics, a random variable is defined by a visible concept, which is represented by a Hamilton matrix H. That means it has a spectral decomposition of the real eigenvalues. In other words, it can create Eq. (4) as shown in the quantum state vector $\left|\uppsi \right.\rangle$, expressed in eigenstate $\left|1\right.\rangle$, $\left|2\right.\rangle$, …, $\left|n\right.\rangle$, the ${c}_{1}$, ${c}_{2}$, …, ${c}_{n}$ corresponds to the complex amplitude:

5 $$\left|\psi \right.\rangle ={c}_{1}\left|1\right.\rangle +{c}_{2}\left|2\right.\rangle +\cdot \cdot \cdot +{c}_{n}\left|n\right.\rangle$$

If the eigenvalue O_1_ O_2_…, O_n_ corresponds to the measured value O of all observable values, then we can measure the result of this random variable by applying the Born rule, and the corresponding probability becomes:

6 $$\mathit{Pr}({O}_{N})={\left|\langle n|\psi \rangle \right|}^{2}={\left|{c}_{n}\right|}^{2}$$

Use the Born rule to convert the classical probability into complex amplitude:

7 $$\mathit{Pr}(A)={\left|{e}^{i{\theta }_{A}}{\psi }_{A}\right|}^{2}\to {\psi }_{A}={e}^{-i{\theta }_{A}}\sqrt{\mathit{Pr}(A)}$$

The full joint probability distribution of quantum can be defined in the same way as in the classical case, with two major differences: (1) the real probability values are replaced by complex probability amplitudes; (2) the probability value is given by the square of the magnitude of the projection. In this sense, it is the quantum full joint complex probability amplitude distribution over N random variables: $\psi ({X}_{1},{X}_{2},...{X}_{N})$, it corresponds to the probability distribution of all of these random variables occurring simultaneously in the Hilbert space, namely, the full joint complex probability amplitude distribution of QLB Network is:

8 $$\psi (X_{1} ,X_{2} , \ldots ,X_{N} ) = \prod\limits_{{j = 1}}^{N} {\psi \left( {X_{j} |Parents\left( {X_{j} } \right)} \right)}$$

In the above equation, X_i_ is a random variable (or network node), $Parents({X}_{i})$ are the Parents of all ${X}_{1}$ nodes, $\psi ({X}_{i})$ is the complex probability amplitude of random variable ${X}_{i}$. The probability values are extracted by applying the Bonn rule, i.e., the square of the joint probability amplitude:

9 $$\mathit{Pr}({X}_{1},...,{X}_{N})={\left|\psi ({X}_{1},...,{X}_{N})\right|}^{2}$$

The quantum marginal probability of discrete random variables can be defined by Formula (8), and the obtained fraction is normalized:

10 $$\mathit{Pr}(X\left|e\right)=\sigma {\left|{\sum }_{y}{\prod }_{k=1}^{N}\psi \left({X}_{k}|Parents\left({X}_{k}\right),e,y\right)\right|}^{2}$$

Based on the expansion of the above formula, the quantum marginalization formula (11) is obtained, which is composed of two parts: the first part represents classical probability, and the second part represents quantum entanglement term, it can be expressed by Eq. (12):

11 $$\mathit{Pr}(X\left|e\right)=\alpha {{\sum }_{i=1}^{\left|Y\right|}\left|{\prod }_{k}^{N}\psi \left({X}_{k}|Parents\left({X}_{k}\right),e,y=i\right)\right|}^{2}+2\cdot Interference$$

12 $$Interferencr={\sum }_{i=1}^{\left|Y\right|-1}{\sum }_{j=i+1}^{\left|Y\right|}\left|{\prod }_{k}^{N}\psi \left({X}_{k}|Parents\left({X}_{k}\right),e,y=i\right)\right|\cdot \left|{\prod }_{k}^{N}\psi \left({X}_{k}|Parents\left({X}_{k}\right),e,y=j\right)\right|\cdot \mathit{cos}({\theta }_{i}-{\theta }_{j})$$

In the above formula, if ${\theta }_{i}-{\theta }_{j}=\pi /2$, then $cos({\theta }_{i}-{\theta }_{j})=0$,it means that the quantum interference term is cancelled and the QLB network collapses to a CBM. In other words, we can regard the QLB Network as a more general and more abstract model of the classical network, because it represents both classical behaviors and quantum behaviors. For normalization purposes, we assume that the decision-maker is subjected to the same quantum interference terms, that is, $({\theta }_{i}-{\theta }_{j})=\theta$. If $cos\theta =1$, then $\theta =0+2k\pi ,k\in Z$, it is equivalent to the maximum constructive interference that quantum probabilistic reasoning can achieve. Similarly, if $cos\theta =-1$, then $\theta =\pi +2k\pi ,k\in Z$, minimal destructive interference is achieved. When $\theta \in [0,\pi ]$, the probability inference calculated using quantum probability theory can have all possible probability values in different ranges. So, the value of $\theta$ represents uncertainty in the decision-making process.

**Figure 1:**
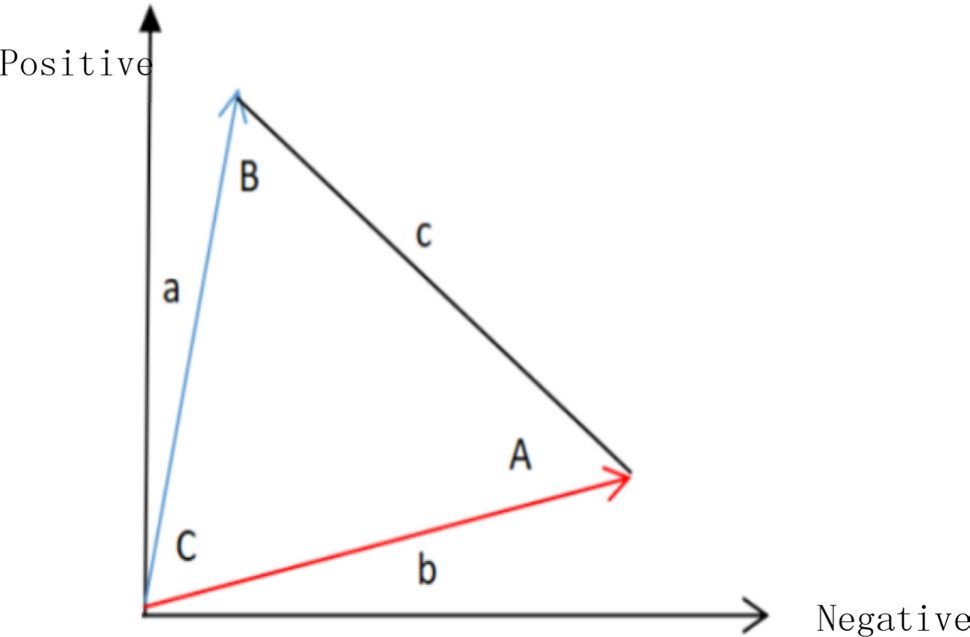
Two vectors represent a particular state. Image source: Drawn with WPS software (Version: 11.1.0.10134. URL: http://www.wps.cn/).

Catarina proposed a heuristic^49^ to construct two column vectors, he proposed the QLB Network only supports binary random variables, that is to say, it has yes or no answer to the query executed by the network. One vector corresponds to the probability that a query against a random variable returns a positive answer, and the other corresponds to the probability that a query against a random variable returns a negative answer. In geometric space, these vectors are shown in Fig. 1. From these two vectors, we can calculate the similarity, such as the angle between the vectors or the distance between them.

The goal of the similarity heuristic is to determine the angle between vector a and vector b that can be used as an equation with parameters θ. In addition, vector c is obtained by calculating the Euclidean distance between vector a and vector b. By comparing the similarity between two vectors, we can infer the hidden property of the participant's belief/behavior from the visible information. Pothos et al.^48^ represent a person's belief/behavior in an N-dimensional vector space, and the similarity between these vectors is measured by a projection operator, which corresponds to the calculation of the square of the length of the projected vector.

We use the law of cosines to determine the angle between vectors, because we only know the length of vector c, so we need to use the law of cosines to compare the similarity between vectors. Catarina proposed a similarity measurement $\Phi$^49^, it represents the similarity between the additional information obtained by processing the original vector. As shown in Eq. (13):

13 $$\begin{aligned} & \left\|c\right\| = \left\|a\right\| - \left\|b\right\| \\ & \theta_{A} = \cos^{ - 1} \left( {\frac{{\left\|b\right\|^{2} - \left\|a\right\|^{2} + \left\|c\right\|^{2} }}{2 \left\|c\right\| \cdot \left\| b\right\|}} \right) \\ & \theta_{B} = \cos^{ - 1} \left( {\frac{{\left\|a\right\|^{2} - \left\|b\right\|^{2} + \left\|c\right\|^{2} }}{2 \left\|c\right\| \cdot \left\|a\right\|}} \right) \\ & \theta_{C} = \cos^{ - 1} \left( {\frac{{\left\|a\right\|^{2} + \left\|b\right\|^{2} - \left\|c\right\|^{2} }}{2\left\|a\right\| \cdot \left\|b\right\|}} \right) \\ & \Phi = \frac{{\theta_{C} - \theta_{B} }}{{\theta_{A} }} \\ & h\left( {a,b} \right) = \left\{ {\begin{array}{*{20}l} \pi \hfill & {(if\;\Phi < 0)} \hfill \\ {\pi - \frac{{\theta_{c} }}{2}} \hfill & {\left( {if\;\Phi > 0.2} \right) } \hfill \\ {\pi - \theta_{c} } \hfill & {\left( {otherwise} \right)} \hfill \\ \end{array} } \right. \\ \end{aligned}$$

## Case study

### Case 1—whether pedestrians cross the street

**Figure 2:**
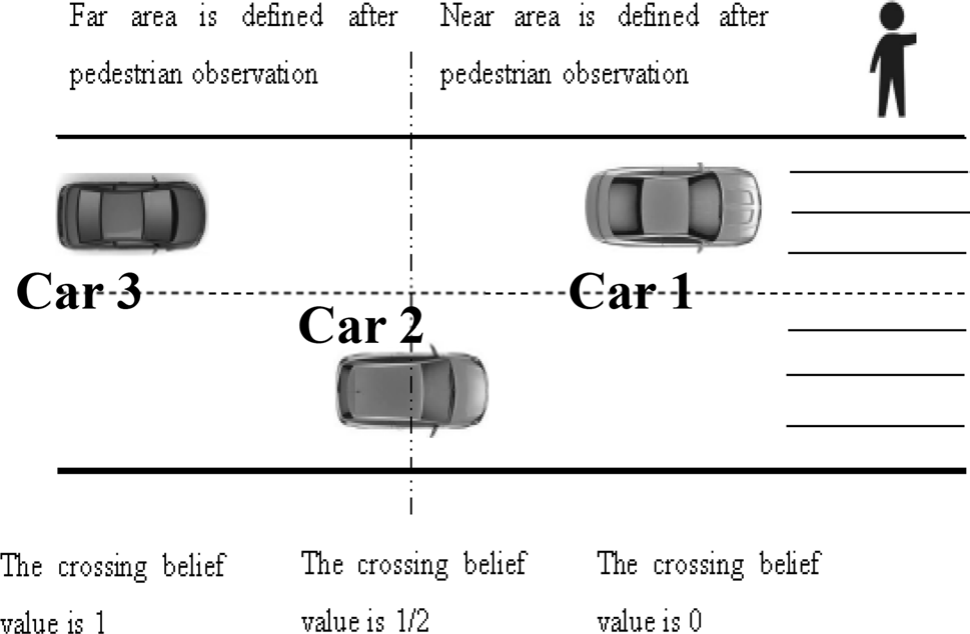
The interaction scene between the autonomous vehicle and the pedestrian when passing the crosswalk. Image source: Drawn with WPS software (Version: 11.1.0.10134. URL: http://www.wps.cn/).

Pedestrians crossing the street scenes are shown in Fig. 2 of this article, pedestrians through the zebra crossing two lanes, the intersection without traffic lights, assume that the three cars in the figure are all autonomous vehicles, Car 1 on the left is the furthest vehicle in the pedestrian's line of sight, assume that the pedestrian can pass the crosswalk with 100% confidence under the condition of Car 1 driving at a constant speed, i.e., the belief value is 1; Similarly, it is assumed that the pedestrian has no confidence to cross the crosswalk at a constant speed in Car 3, i.e., the belief value is 0; assume that Car 2 is at this point halfway between a belief value of 1 and 0, i.e., a belief value of 0.5.

#### Classical Bayesian Model (CBM)—Intent estimation of pedestrian crossing

This paper uses Matlab as a simulation platform, assuming independent events: pedestrians crossing the street when a vehicle is approaching from far area, pedestrians crossing the street when a vehicle is approaching from near area. Hypothetical variable: the belief value of pedestrian cross the street is 1/2–1 when observing the vehicles is in far area (represented by the abscissa Y), the belief value of pedestrian cross the street is 0–1/2 when observing the vehicles is in near area (represented by the abscissa Z).

**Figure 3:**
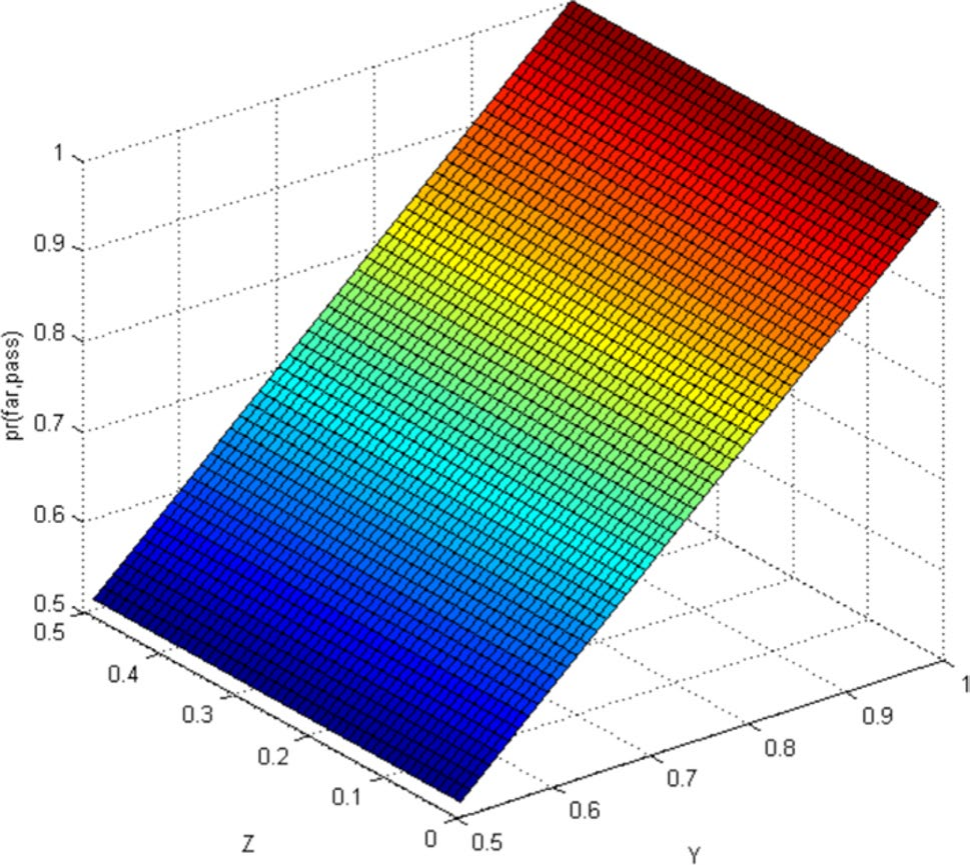
Probability distribution of pedestrians choosing to cross the street when autonomous vehicle is in far area (CBM). Image source: Drawn with MATLAB software (Version: MATLAB R2018a. URL: www.mathworks.com/).

**Figure 4:**
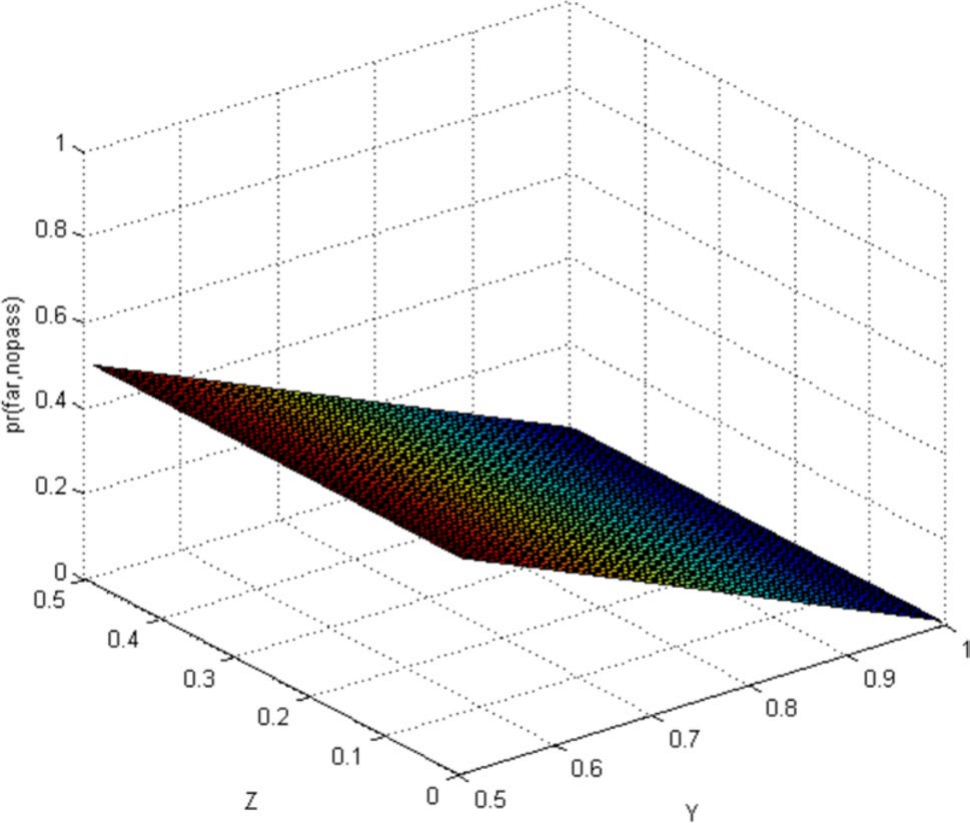
Probability distribution of pedestrians choosing to miss the street when autonomous vehicle is in far area (CBM). Image source: Drawn with MATLAB software (Version: MATLAB R2018a. URL: www.mathworks.com/).

**Figure 5:**
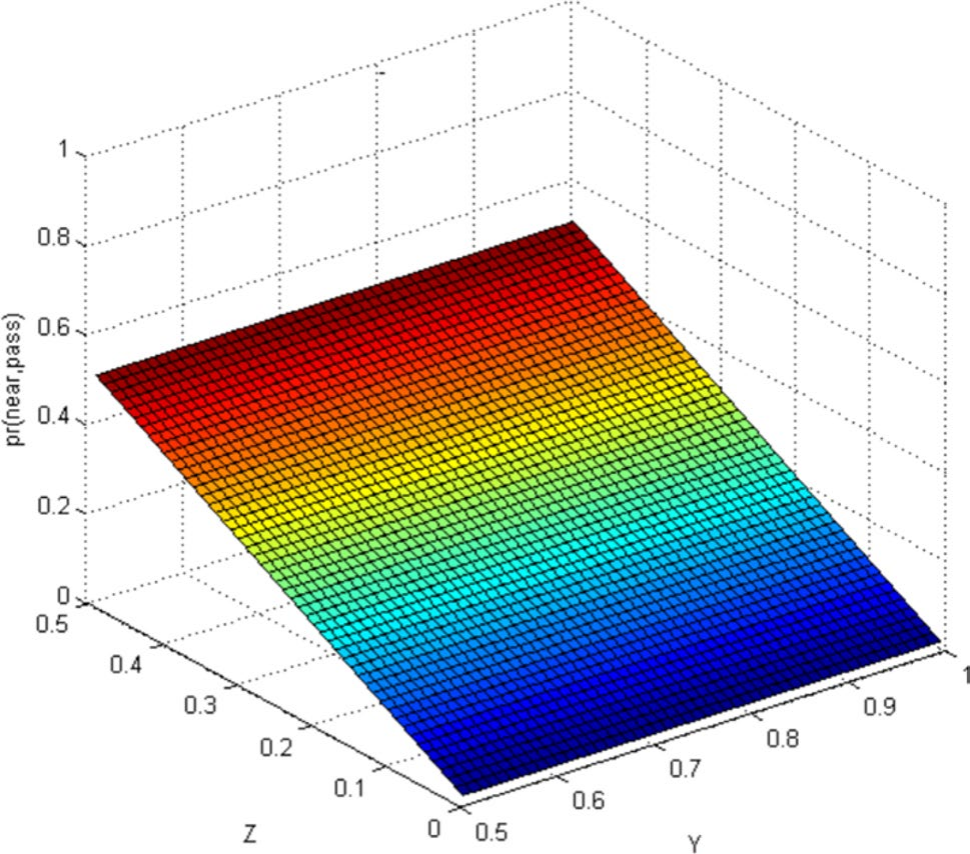
Probability distribution of pedestrians choosing to cross the street when autonomous vehicle is in near area (CBM). Image source: Drawn with MATLAB software (Version: MATLAB R2018a. URL: www.mathworks.com/).

**Figure 6:**
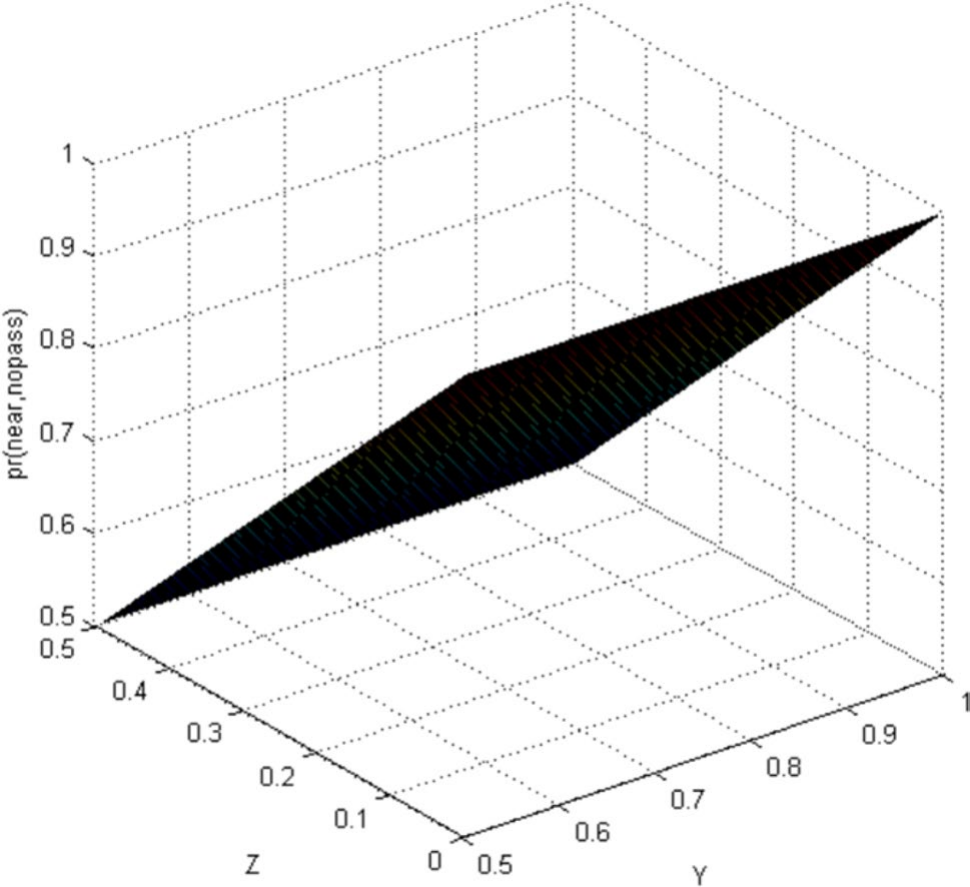
Probability distribution of pedestrians choosing to miss the street when autonomous vehicle is in near area (CBM). Image source: Drawn with MATLAB software (Version: MATLAB R2018a URL: www.mathworks.com/).

According to the CBM, when the pedestrian observes the autonomous vehicle with far area, the situation in the near area is not taken into account. Similarly, the situation is the same when pedestrians observe the autonomous vehicle in near area. Therefore, the results of CBM simulation can distinguish the two situations of far area and near area (Figs. 3, 4, 5, 6). In the real world, the estimation of pedestrian's intention when crossing the street is often based on observing all the surrounding environment, and the calculation using the CBM obviously does not accord with the actual situation.

#### Quantum-like Bayesian Model (QLB)—Intent estimation of pedestrian crossing

**Figure 7:**
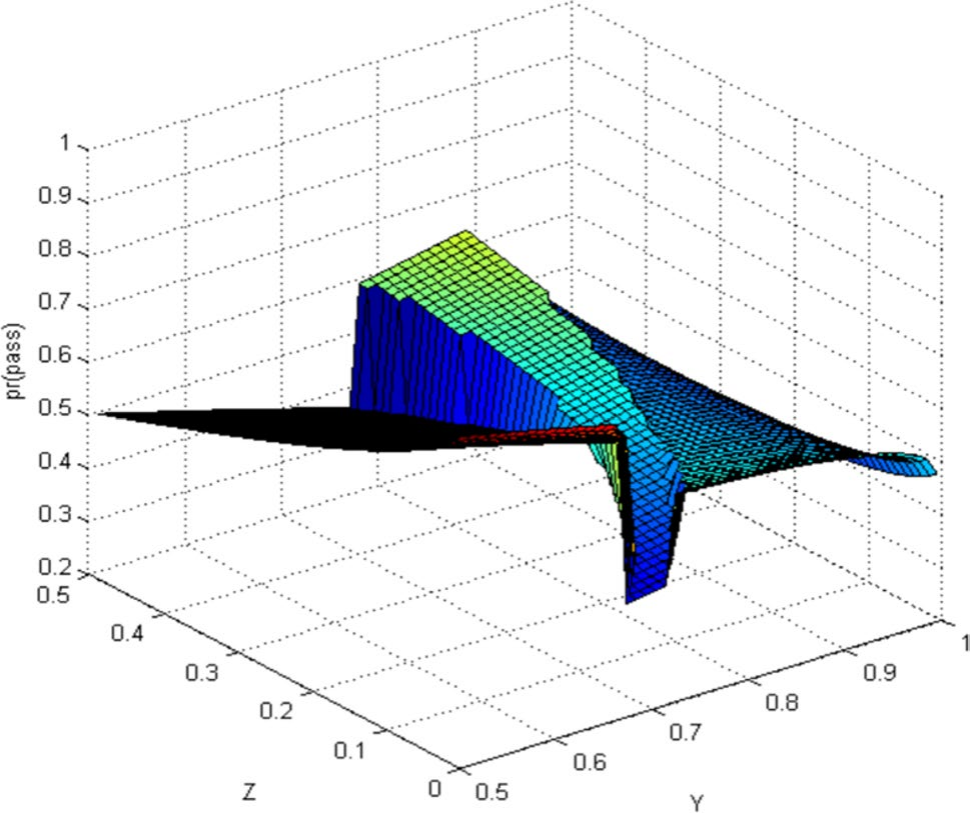
Probability distribution of pedestrians choosing to cross the street (quantum Bayesian model). Image source: Drawn with MATLAB software (Version: MATLAB R2018a. URL: www.mathworks.com/).

**Figure 8:**
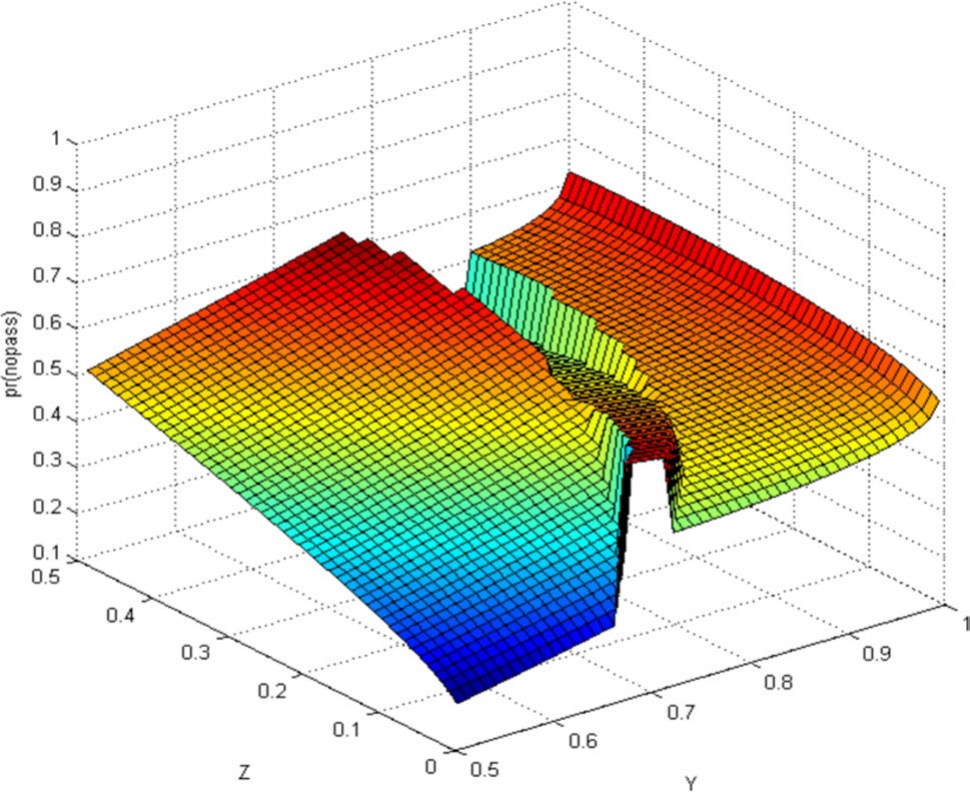
Probability distribution of pedestrians choosing to miss the street (quantum Bayesian model). Image source: Drawn with MATLAB software (Version: MATLAB R2018a. URL: www.mathworks.com/).

When using QLB to calculate, pedestrians observe the autonomous vehicle with far while taking into account the situation of the autonomous vehicle with near. Similarly, the situation is the same when pedestrians observe the autonomous vehicle with near. The simulation results are shown in Figs. 7 and 8.

By comparing the CBM with QLB, it can be clearly seen that the probability distribution simulated by the CBM is a plane. As the distance between pedestrians and autonomous vehicles gets closer and closer, the probability of pedestrians crossing the street becomes smaller and smaller. Although this is consistent with common sense, it does not correspond to the actual behavior of human beings. And QLB simulate the probability distribution is irregular surface, this is due to human in addition to do the action according to the classical probability will produce irrational behavior, irrational behavior led to the formation of quantum interference item to join, so more specific QLB reflects the pedestrian behavior probability when interacting with autonomous vehicles.

QLB takes the more comprehensive situation into account and explains the influence of irrational pedestrian behavior on the intention estimation of autonomous vehicles. The autonomous vehicles can more accurately estimate whether pedestrians are crossing the street through the QLB.

### Case 2—Pedestrian crossing track prediction

The movement characteristics of pedestrians and the behavioral characteristics of pedestrians when crossing the street are complex and changeable. In the face of an approaching car, pedestrians may take different actions, such as speeding forward or stopping avoid collision^2^, and even a series of irrational behaviors such as answering the phone or being attracted by other things to divert their attention. It is relatively easy for a human driver to judge a pedestrian's intentions based on experience, and then choose to continue to drive or to slow down to give way, and in the automated driving scenarios, only through the sensor detection and tracking is difficult to draw a pedestrian next trajectory^3^, the uncertainty factors of pedestrian crossing can reduce the transportation efficiency and safety accident frequency. Kitani et al.^50^ have demonstrated that semantic knowledge about static environments (e.g. the position of sidewalks, the expansion of grass area, etc.) helps predict future pedestrian trajectrajeces more accurately than models that ignore scene information.

#### Social-LSTM

Pioneering work^51–53^ proposes methods for modeling human-to-human interactions (often referred to as “social forces”) to improve robustness and accuracy in multi-target tracking problems. However, most of these studies are limited by two assumptions: (1) they use hand-crafted functions to build “interactive” models for specific Settings, rather than passing them around in a data-driven manner. This leads to models that are more inclined to capture simple interactions (such as repulsion/attraction) and may not be suitable for more complex interaction scenarios. (2) They focus on modeling close interactions between people (to avoid direct collisions). However, they did not foresee the interactions that might occur in the more distant future.

**Figure 9:**
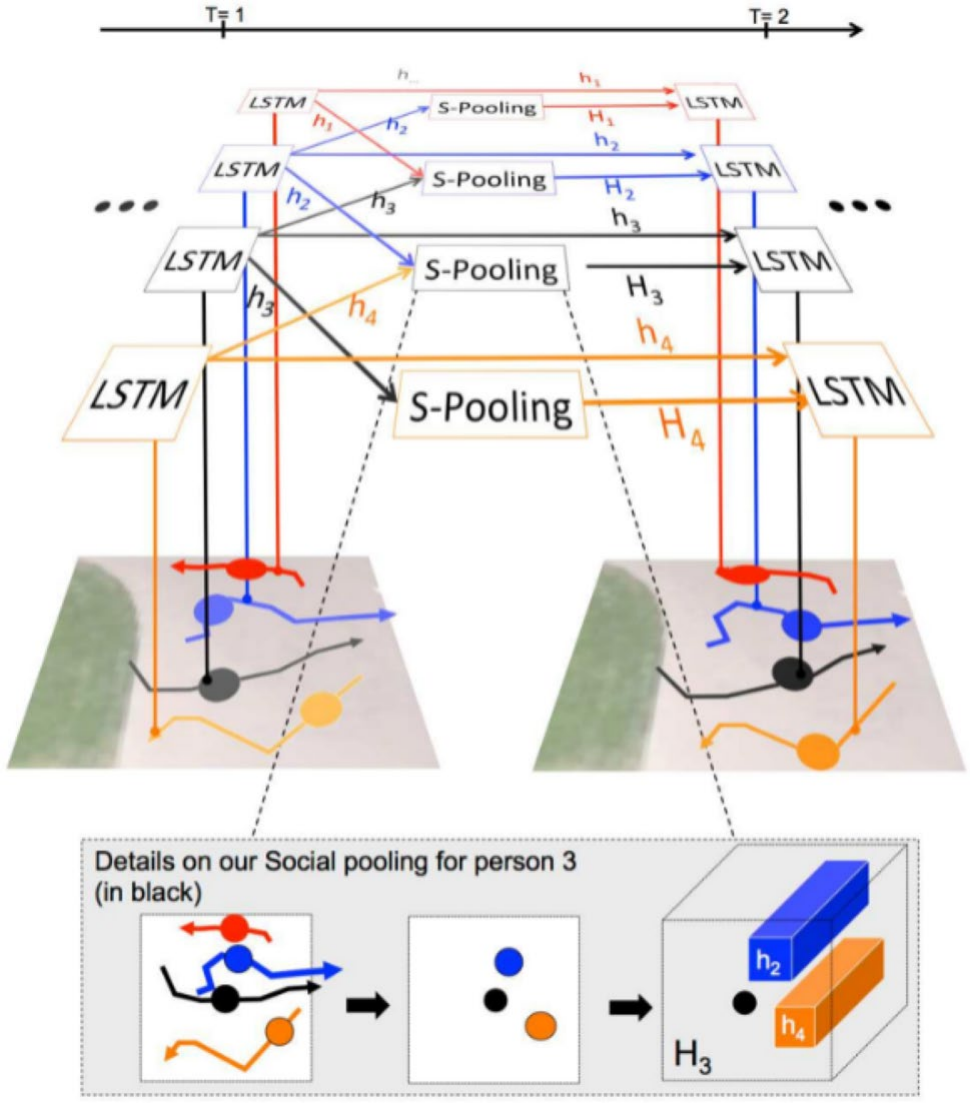
Overview of Social-LSTM method. Use a separate LSTM network for each trajectory in a scene. The LSTMs are then connected to each other through a Social pooling (S-pooling) layer. Unlike the traditional LSTM, this pooling layer allows spatially proximal LSTMs to share information with each other. The bottom row shows the S-pooling for one person in the scene. The hidden-states of all LSTMs within a certain radius are pooled together and used as an input at the next time-step. Image source: Drawn with MATLAB software (Version: MATLAB R2018a. URL: www.mathworks.com/).

Spired by the success of the Long-Short Term Memory Network (LSTM) applied to different sequence prediction tasks, we extended it to human trajectory prediction. Although LSTMs have the ability to learn and copy long sequences, they do not capture dependencies between multiple related sequences. This problem is solved by a new architecture that connects the LSTMs corresponding to adjacent sequences^54^. A “social” pooling layer has been introduced that allows LSTMs of spatial proximal sequences to share their hidden state with each other. This architecture, called “social-LSTM”, can automatically learn typical interactions between tracks that overlap in time (Fig. 9). The model uses the existing dataset of human trajectories without the need for any additional annotations to learn the common-sense rules and conventions that humans observe in social Spaces.

#### A social force model with QLB optimization parameters

The parameter values required by the classical social force model are all obtained by model calibration method. According to the existing studies^55^, the labeled results are listed in Table 1.

**Table 1:** Parameter calibration results of classical social force model.

Parameters	Value
The force intensity of other pedestrians on pedestrians	0.42
The force intensity of the vehicle on the pedestrian	0.71

After above analysis, the classic social force model has obvious unsuitable used in complex traffic scenes, but the QLB can “update” the manually calibrated parameter values, after “update” the values of the parameters due to considering the influence of irrational joined the quantum interference items, such as pedestrians crossing the street to see other pedestrians running sudden acceleration, change the original force strength. The following figure shows the Variation law of the “update” of the QLB when the intensity of action changes with the classical probability calibration.

**Figure 10:**
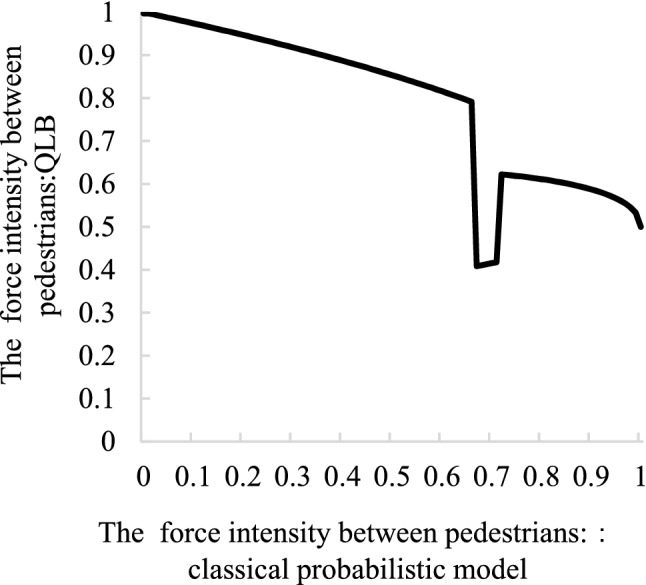
Variation law of force intensity between pedestrians before and after updating. Image source: Drawn with WPS software (Version: 11.1.0.10134. URL: http://www.wps.cn/).

**Figure 11:**
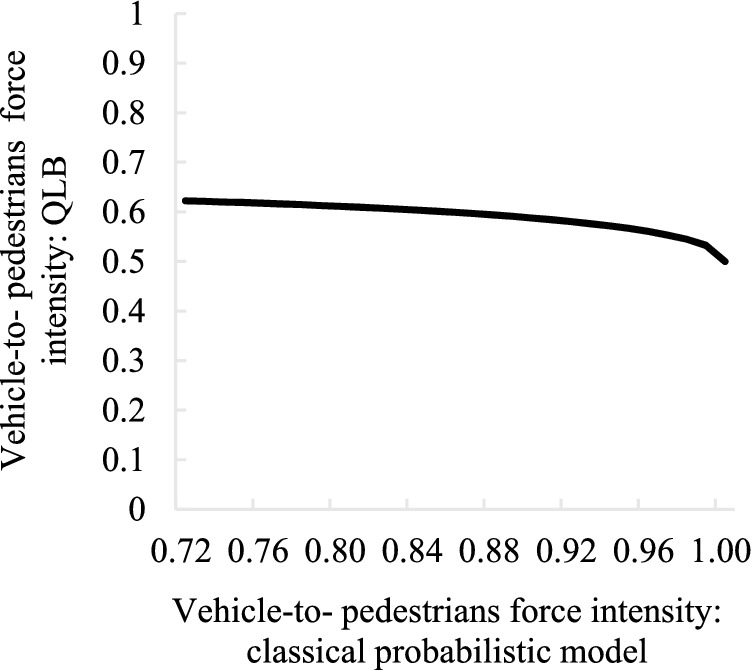
Variation law of vehicle–pedestrian interaction intensity before and after updating. Image source: Drawn with WPS software (Version: 11.1.0.10134. URL: http://www.wps.cn/).

The abscissa represents the value of the original parameter with classical probability. In Fig. 10, as the impact intensity of other pedestrians on pedestrians gradually increases from 0, the new impact intensity (vertical axis) obtained by the “update” of QLB in the range of 0.67–0.72, it first decreased and then increased, which is caused by the change of the similarity measurement value. The calibration of the original parameter value under the classical probability is related to the occupancy and movement speed of pedestrians. In the real world, when pedestrians see other pedestrians moving very slowly or occupying very little space, they will increase their vigilance to prevent irrational behaviors. Therefore, the initial value of the “updated” effect intensity in the QLB is higher. In Fig. 11, parameters larger than the calibration value of the classical social force model are considered in the “update” of the intensity of the vehicle's effect on pedestrians. It can be seen that the intensity of the vehicle's effect on pedestrians after the “update” is above 0.5, which is smaller than the original parameter and is consistent with the fact that the vehicle often proactively accommodates pedestrians in the real world.

## Experimental analysis

In the above two cases, the more complex case 2 is selected as the experiment of this study to illustrate the advantages of QLB proposed in this paper. CMB has been proved to be unsuitable for complex traffic scenarios. In this section, the data-driven social-LSTM model and QLB social-force model with optimized parameters are compared through data set experiments to analyze the characteristics of the two methods.

**Figure 12:**
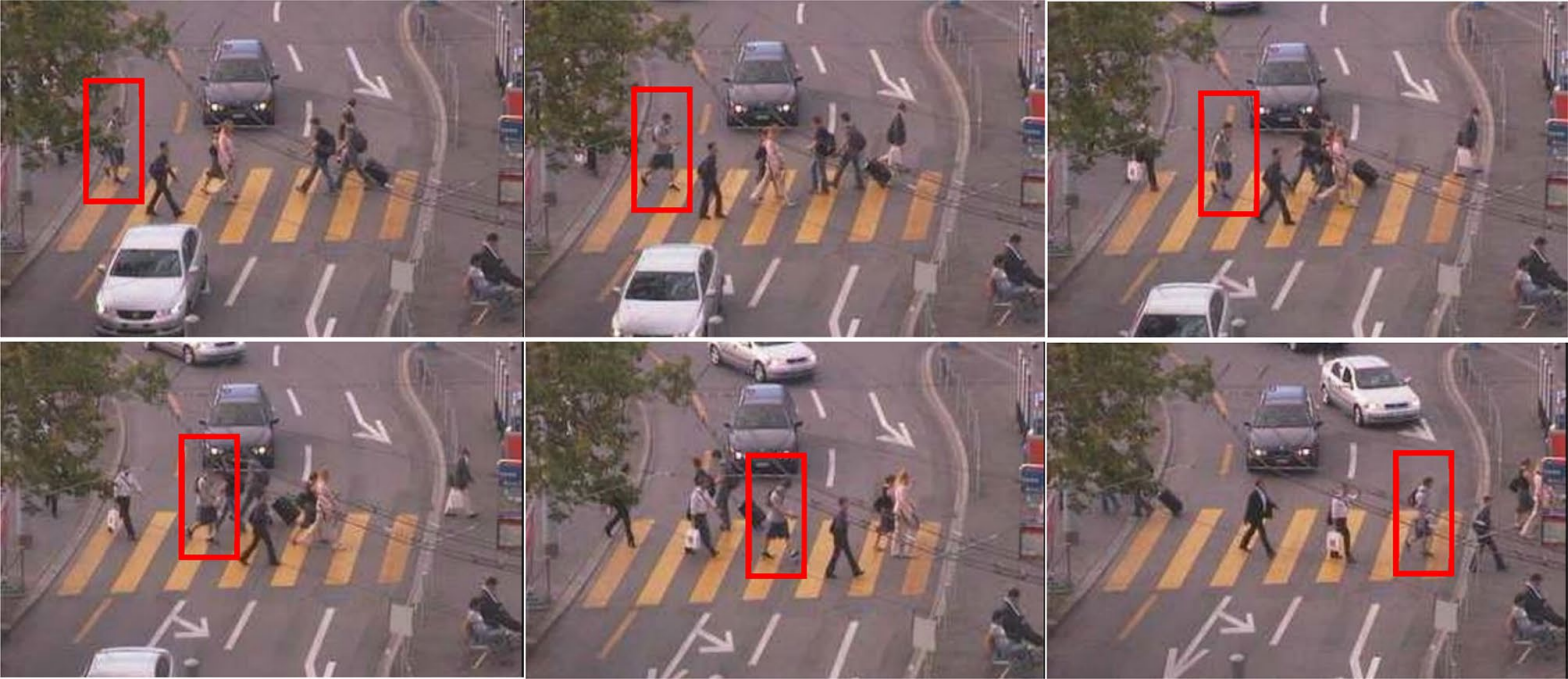
Pedestrian crossing data set. Image source: University of California-Berkeley, *International interactive motion data set*. USA: University of California. http://interaction-dataset.com (2020).

**Figure 13:**
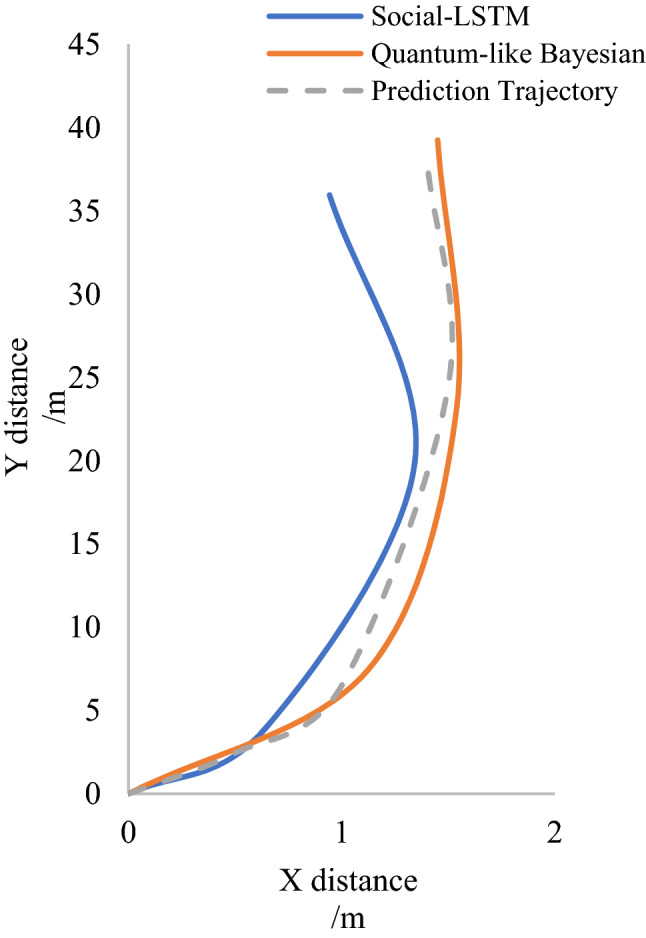
Comparison of trajectories predicted by the Social LSTM model and QLB with observed data. Image source: Drawn with WPS software (Version: 11.1.0.10134. URL: http://www.wps.cn/).

The data set required for the experiment was obtained from the interactive data set website^56^ (Fig. 12), and the trajectories predicted by the social-LSTM model and QLB were compared with the observed data respectively (Fig. 13). Pick up one from the pedestrian as forecaster (red frame), as set intervals delta ${\Delta }_{t}=1s$. As shown in the figure below.

By analogy, the trajectories of multiple pedestrians are predicted in this data set^56^, and the average deviation errors predicted by the two models are obtained. As shown in the table below (Table 2).

**Table 2:** Comparison of Social-LSTM and QLB average displacement error.

Methods	Social-LSTM^54^	Our Quantum-like Bayesian
Average displacement error	0.2	**0.11**

Significant values are in [bold].

It can be seen from the trajectory prediction results of a single pedestrian and the predicted average displacement errors of multiple pedestrians that the quantum-like Bayesian model QLB is superior to the Social-LSTM model for the following reasons: (1) the Social-LSTM model is applicable to the situation that humans obey common-sense rules and does not consider the occurrence of irrational behaviors. Therefore, in the case of irrational behaviors among pedestrians or between pedestrians and vehicles in the data set, the prediction ability will be reduced; (2) Social-LSTM model relies on data-driven, while edge events are not included in these massive data. QLB can take all the surroundings into account due to their physical significance and explain edge events that data-driven model cannot. (3) QLB does not adhere to any assumptions and considers all situations during interaction, while social-LSTM model assumes constant speed in some interaction processes^54^, which is not in line with the actual situation.

In order to evaluate the ability of quantum-like Bayes method in pedestrian trajectory prediction more comprehensively, this paper compares our method with nine of the most advanced methods in the past four years. Vanilla LSTM^57^, social-LSTm^54^, SGAN^58^, Sophie^59^, PITF^60^, social-biga T^61^, social-stgCNN^62^, RSGB^63^ and STAR^64^.

The results are shown in Table 3, evaluated using ADE (average displacement error) and FDE (final displacement error) metrics. The proposed approach is evaluated in public ETH^65^ and UCY^66^. The two datasets contain five sub datasets, namely ETH, HOTEL, UNIV, ZARA1 and ZARA2. All sub datasets contain real pedestrian tracks with rich human-to-human object interaction scenarios, including scenarios where people cross each other, form and disperse groups, as well as avoiding collisions.

**Table 3:** Comparison with the baselines approach on the public benchmark dataset ETH and UCY for ADE/FDE. Our QLB significantly outperform the comparison state-of-the-art works. The lower the better.

Model	Year	ETH	HOTEL	UNIV	ZARA1	ZARA2	AVG
Vanilla-LSTM^57^	2016	1.09/2.41	0.86/1.91	0.61/1.31	0.41/0.88	0.52/1.11	0.70/1.52
Social-LSTM^54^	2016	1.09/2.35	0.79/1.76	0.67/1.40	0.47/1.00	0.56/1.17	0.72/1.54
SGAN^58^	2018	0.87/1.62	0.67/1.37	0.76/1.52	0.35/0.68	0.42/0.84	0.61/1.21
Sophie^59^	2019	0.70/1.43	0.76/1.67	0.54/1.24	0.30/0.63	0.38/0.78	0.51/1.15
PITF^60^	2019	0.73/1.65	0.30/0.59	0.60/1.27	0.38/0.81	0.31/0.68	0.46/1.00
Social-BIGAT^61^	2019	0.69/1.29	0.49/1.01	0.55/1.32	0.30/0.62	0.36/0.75	0.48/1.00
Social-StgCNN^62^	2020	0.64/1.11	0.49/0.85	0.44/0.79	0.34/**0.53**	0.30/0.48	0.44/0.75
RSBG^63^	2020	0.80/1.53	0.33/0.64	0.59/1.25	0.40/0.86	0.30/0.65	0.48/0.99
STAR^64^	2020	**0.56**/1.11	**0.26/0.50**	0.52/1.15	0.41/0.90	0.31/0.71	0.41/0.87
Our QLB	–	**0.56/1.02**	**0.26/**0.51	**0.35/0.72**	**0.29/0.53**	**0.26/0.43**	**0.32/0.63**

Significant values are in [bold].

The results show that proposed method is significantly s better than other methods in both ETH and UCY data sets. In terms of ADE measurement, our method is also better than the previous best method STAR^64^, and in terms of FDE measurement, our method is superior to the previous best method social-stgCNN^62^.

It is widely known that the underlying reason, as that our approach can take the influence of environmental factors on the target into account. Interestingly, our method is s better than all methods based on intensive interaction for UNIV sequences with dense crowd scenes, such as SGAN^58^, Sophie^59^, social-biga T^61^, social-stgCNN^62^ and STAR^64^.

It is speculated that the approach based on quantum cognitive theory may capture redundant interaction objects, leading to prediction errors. The difference is that our method can consider the influence of environmental factors on the object, being conducive to better performance.

## Conclusion and prospect

A model based on quantum cognitive theory is presented, which can take irrational behaviors and marginal events into account when judging whether pedestrians are crossing the street or not and when predicting pedestrian crossing trajectory. In the case of judging whether pedestrians cross the street, we compare QLB with CBM, and clearly conclude that QLB can take the irrational factors in the interaction process into consideration, reflecting more real pedestrian crossing intention; in the case of pedestrian crossing trajectory prediction, QLB is used to update the force intensity parameters in the classical social force model to reflect a more real interactive environment. The proposed method is better than current advanced data-driven methods (Social-LSTM) in terms of open data sets. It also outperformed nine major approaches, and is more advantageous in explaining the uncertain and irrational behaviors as well as interactions of other traffic participants, being helpful for the more comprehensive analysis and estimation of the future intentions and actions of pedestrians by autonomous vehicles.

Quantum-like Bayes (QLB) method has a good effect on pedestrian prediction, but it has some limitations. For example, further optimization is needed in terms of interpretation, and a more perfect QLB model will be established in combination with knowledge Graph in the future research work. Meanwhile, in the process of simulation, the Internet of vehicles technology is combined with the roadside unit data, requiring more comprehensive environmental information. The author believe that more comprehensive analysis results will be obtained to further explore the interaction with other traffic participants in autonomous driving.

Based on the quantum cognitive theory, this paper conducts applied research on the simple pedestrian crossing, but the research methods adopted are also instructive and referential for more and more complex scenes in automatic driving. This paper is the first attempt to apply quantum cognitive theory into autonomous driving, and it provides a new reference for the study of human traffic participants bounded rational behavior intention cognition. In future task, more complex traffic scenes will be used to explore the interaction between autonomous driving and other traffic participants through the combination of quantum theory and deep learning.
